# Dynamic Voltage-Response Estimation for Blade-Tip Timing Sensors Based on EHHO-BP Network and Waveform Mapping

**DOI:** 10.3390/s26165236

**Published:** 2026-08-18

**Authors:** Wei Huang, Liang Zhang, Qingkai Xu, Han Wu, Long Chen

**Affiliations:** 1 Faculty of Mechanical Engineering and Automation, Liaoning University of Technology, Jinzhou 121001, China; 17375755194@163.com (W.H.); xu159357789123@163.com (Q.X.); wh287289@163.com (H.W.); 19834730781@163.com (L.C.); 2 Key Laboratory of Vibration and Control of Aero-Propulsion System, Ministry of Education, Northeastern University, Shenyang 110819, China

**Keywords:** blade-tip timing sensor, static calibration, EHHO-BP model, waveform mapping, blade vibration

## Abstract

Blade-tip timing (BTT) sensor technology is widely used for non-contact blade vibration measurement. However, conventional BTT methods mainly rely on sparse time-of-arrival (TOA) information, which limits continuous sensor-domain characterization of blade vibration. To address this limitation, this paper proposes a dynamic voltage-response estimation framework combining an Elite Harris Hawks Optimization-based backpropagation neural network (EHHO-BP) with waveform mapping. Zero-speed static calibration experiments are used to establish the nonlinear relationships among blade-tip radial clearance, relative angular position, and sensor voltage, and the EHHO-BP model is employed to estimate the calibration response. Blade vibration displacement obtained from numerical models or reconstructed from BTT TOA measurements is then mapped to a continuous dynamic voltage response under a quasi-static transfer assumption. Single-harmonic and multi-harmonic simulations demonstrate the response-estimation process. In the static calibration comparison, EHHO-BP achieves a median RMSE of 8.92 mV, CVRMSE of 0.56%, MAE of 6.34 mV, and R^2^ of 0.99982. Rotating experiments at 400, 600, 800, 1200, and 1500 rpm further evaluate the transferability of the zero-speed calibration model without retraining or speed-dependent correction. Across the tested speed range, the correlation coefficient remains between 0.9868 and 0.9989 and R^2^ remains between 0.952 and 0.997; however, the FWHM error increases from 1.469% at 400 rpm to 22.662% at 1500 rpm. These results demonstrate the good transferability of the proposed quasi-static mapping at low-to-moderate rotational speeds while revealing a progressive deterioration in temporal waveform consistency at higher speeds. The proposed framework, therefore, provides a continuous sensor-domain representation of BTT-derived blade vibration displacement and an experimentally supported assessment of its speed-dependent applicability.

## 1. Introduction

Turbine blades utilized in gas turbines, compressors, and aeroengine units maintain uninterrupted operation under rigorous thermal and pressure conditions, with their dynamic behavior directly governing the comprehensive performance and safety stability of the corresponding machinery [1]. High-cycle fatigue and crack propagation induced by blade vibration further emerge as critical factors jeopardizing structural integrity and service life [2]. Thus, accurately tracking the vibration behaviors of blades is essential to achieving preventive maintenance and optimizing design.

Traditional contact monitoring relies primarily on strain gauges, yet its application is restricted by intrusiveness, high costs, and inadequate durability in harsh environments [3]. BTT technology has attracted widespread attention in academic circles because it enables the synchronized monitoring of multiple blades by measuring the TOA of the blade tips without requiring physical contact and has attracted substantial academic and industrial attention [4]. However, conventional BTT methods mainly rely on sparse TOA information [5,6], which limits the acquisition of continuous blade vibration responses. The limited measurement information leads to sparse sampling, noise sensitivity [7], and potential frequency-aliasing problems [8], motivating the development of advanced BTT signal-processing methods.

To overcome the information loss inherent in conventional TOA-based BTT measurements, considerable research has focused on improving vibration reconstruction from nonuniform and undersampled blade-tip observations. Xu et al. [9] formulated the recovery of undersampled BTT signals as a sparse-reconstruction problem and introduced generalized minimax-concave regularization to improve the estimation of blade vibration characteristics. Liu et al. [10] employed the Multiple Signal Classification (MUSIC) algorithm to reconstruct blade-tip vibration signals and identify vibration parameters from limited BTT samples. From the perspective of sampling characteristics, Yang et al. [11] systematically investigated the aliasing-frequency distribution associated with high-resolution BTT sampling, revealing the strong dependence of frequency identification on the spatially sparse and nonuniform sampling configuration. These methods have substantially improved the ability of conventional BTT systems to extract vibration frequency, amplitude, and phase information from sparse measurements. Nevertheless, their principal objective remains the reconstruction or parameter identification of blade vibration from discrete TOA-derived displacement samples, while most of the voltage information contained in the complete blade-passing pulse is discarded after the arrival time has been extracted.

To exploit this additional sensor-domain information, increasing attention has been devoted to full-waveform BTT analysis. Heller et al. [12] demonstrated that blade vibration produces measurable modulation of BTT and tip-clearance sensor waveforms and developed an optimization-based method for identifying single- and multi-harmonic vibration parameters directly from complete sensor waveforms. Chen et al. [13] subsequently proposed an interpolated wideband signal reconstruction method to recover blade-tip signals using prior information on the signal bandwidth and center frequency. Bian et al. [14] developed the Probe Waveform Spectrum Reconstruction (PWSR) method, which extracts additional displacement information from the probe output waveform and combines it with signal reconstruction to reduce the dependence of vibration identification on the number and arrangement of BTT probes. Zhang et al. [15] further established a waveform-mapping approach between statically calibrated sensor waveforms and dynamically measured blade-passing waveforms, demonstrating that the variation in the complete sensor pulse can be used for blade-vibration parameter identification. More recently, Bian et al. [16] extracted both displacement and velocity information from the full pulse waveform of a single sensor for blade monitoring under non-stationary operating conditions. Huang et al. [17,18] further incorporated multiple vibration-related information sources into sparse-reconstruction and dual-sensor BTT frameworks, while full-waveform parameter identification using dual sensors has also been investigated for complex blade vibration conditions [19]. These developments demonstrate that the sensor waveform contains useful spatial and kinematic information beyond a single TOA value and provide an important basis for waveform-level analysis of BTT measurements.

In parallel with vibration reconstruction and full-waveform parameter identification, several studies have investigated the prediction of the BTT sensor waveform itself from static calibration data. Zhang et al. [20] established a two-dimensional relationship among blade-tip clearance, relative blade position, and sensor output voltage using static calibration experiments and employed a Kriging surrogate model to predict BTT sensor waveforms at previously unmeasured blade-tip clearances. Their results demonstrated that static calibration data can be used to construct a continuous sensor-response surface and predict waveform variations caused by changes in blade–sensor geometry. Subsequently, Zhang and Chen [21] introduced a radial basis function neural network to approximate the nonlinear relationship contained in the static calibration data and verified the feasibility of data-driven models for BTT waveform prediction. These studies indicate that the voltage response of a BTT sensor can be represented as a nonlinear function of the relative blade-tip position and radial clearance and therefore provide a physical and data-driven basis for estimating sensor responses from blade-motion information.

However, the above studies predominantly address two related but distinct inverse problems. Sparse-reconstruction and full-waveform methods generally infer blade vibration parameters from measured TOA samples or dynamically acquired sensor waveforms, whereas static calibration-based methods mainly predict sensor responses at prescribed geometric positions or blade-tip clearances. Although waveform mapping has demonstrated the relationship between static and dynamic BTT waveforms [15], comparatively limited attention has been devoted to the forward estimation problem in which blade vibration displacement obtained from BTT measurements is first reconstructed continuously and subsequently transformed through a static calibration model to estimate the complete dynamic sensor-voltage response. In particular, the applicability of a voltage–position relationship established under zero-speed calibration to different rotating-speed conditions has not been sufficiently examined. Consequently, whether the instantaneous voltage response during blade passage can be approximated by the corresponding zero-speed static calibration relationship, and over what rotational-speed range such a quasi-static approximation remains valid, constitute important issues for calibration-based dynamic response estimation.

Motivated by these considerations, this study hypothesizes that, within an appropriate operating-speed range, the instantaneous output of the BTT sensor can be approximated by the static voltage–position relationship evaluated at the instantaneous blade-tip position. Based on this quasi-static assumption, an enhanced Harris Hawks optimization-based a BP neural network (EHHO-BP) is first employed to establish the nonlinear mapping among the radial blade-tip clearance, relative blade position, and sensor voltage from zero-speed calibration data. The continuously reconstructed circumferential blade vibration displacement obtained from BTT measurements is then combined with the rigid-body blade-passing trajectory and mapped into the sensor-voltage domain, enabling forward estimation of the continuous dynamic voltage response. Unlike existing approaches that primarily extract vibration parameters from measured full waveforms, the proposed framework focuses on reconstructing a continuous sensor-domain response from BTT-derived blade-motion information. Furthermore, the same zero-speed calibration model is applied without retraining or speed-dependent correction at multiple rotational speeds, allowing for the transferability and operating limitations of the quasi-static mapping assumption to be experimentally evaluated. The key novel contributions of this work are outlined in the following two subsequent aspects:

- Estimation of the static calibration voltage response using an EHHO-optimized BP neural network. To address the limitation of sparse voltage information contained in the static calibration samples, the EHHO is employed to refine the BP neural network. The network weight coefficients and node thresholds are gradually optimized and determined during the iterative process. Combined with the interpolation method, continuous estimation of the static calibration voltage response is achieved. This provides a reliable calibration basis for subsequent dynamic response estimation.
- Dynamic voltage-response estimation for blade vibration is achieved based on static waveform calibration. Blade vibration displacement is associated with the estimated sensor-voltage response through the waveform mapping relationship established by the proposed method. Based on this mapping principle, the dynamic voltage response corresponding to the blade vibration conditions can be estimated. The proposed method provides an approach for estimating continuous voltage-response profiles corresponding to the blade vibration states under the blade vibration conditions and offers additional data support for the blade-vibration response analysis. It can characterize the phase variation characteristics associated with the blade vibration response, and the estimated response waveform can reflect the combined effects of multiple vibration components. Furthermore, it enables continuous estimation of sensor responses from blade-vibration displacement information derived from BTT measurements by incorporating the calibration relationship and vibration displacement model.

The remainder of this paper is organized as follows: Section 2 introduces the static calibration method, EHHO-BP model, and waveform-mapping formulation. Section 3 describes the numerical simulation setup, experimental apparatus, static calibration procedure, and multi-speed validation protocol. Section 4 presents and discusses the static calibration results, dynamic-response simulations, and experimental validation. Finally, Section 5 summarizes the main conclusions, limitations, and future work.

## 2. Theoretical Methods

### 2.1. Sensor-Based Static Calibration Method for Waveform

According to the fundamental principle of electromagnetic induction, an induced current is produced as a blade travels across a magnetic field and cuts magnetic flux lines, which is termed the eddy current effect [22]. When a dedicated blade-tip sensor is employed to obtain BTT data from blades with a fixed thickness, where U denotes the sensor output voltage, c is the radial blade-tip clearance, and α represents the relative angular position between the blade centerline and the sensor. Their relationship is given as follows:

(1) U=F(c,α)

For a prescribed blade-tip clearance of $c=c_{0}$, the two-dimensional static calibration relationship reduces to a one-dimensional voltage–angular-position mapping [21], as follows:

(2) $$U=F(c_{0},\alpha )=f_{c_{0}}(\alpha )$$

where this function, f, is the one fitted to the fixed calibration curve.

Static calibration is performed under zero-speed conditions to establish the relationship between the blade-tip position and sensor voltage. A prescribed radial clearance is set on the static calibration rig, and the sensor output is recorded at a series of relative angular positions. The procedure is repeated for multiple radial clearances from 0.10 to 0.55 mm, producing a family of voltage–position curves. After the model fitting, these measurements form a continuous static calibration relationship that is subsequently used for dynamic waveform mapping. Details of the calibration apparatus and acquisition procedure are provided in Section 3.3.

### 2.2. Method for BP Neural Network Optimization Based on the EHHO Algorithm

BP neural networks have strong nonlinear approximation capability; however, their training performance is sensitive to the initial weights and biases and may be affected by local optima [23]. To improve the estimation accuracy and robustness of the static calibration model, the EHHO algorithm is employed to optimize the initial parameters of the BP neural network before backpropagation training.

In this study, a three-layer feedforward BP network with a 2–6–1 architecture is adopted. The two input variables are the blade-tip radial clearance, c, and the relative angular position, α, and the network output is the processed sensor voltage, U. The hidden layer contains six neurons using the hyperbolic tangent sigmoid transfer function (tansig), whereas the output layer contains one neuron using the linear transfer function (purelin). Before training, the input and output variables are normalized to the interval [−1, 1]. The network output can be expressed as follows:

(3) $$U=W^{\left(2\right)}\tan sig(W^{\left(1\right)}z+b^{\left(1\right)})+b^{\left(2\right)}$$

where

(4) $$z={\left(c,\alpha \right)}^{T}$$

W^(1)^∈R^6×2^ and b^(1)^∈R^6×1^ denote the weights and biases between the input and hidden layers, respectively, while W^(2)^∈R^1×6^ and b^(2)^ denote the weights and bias of the output layer.

To enable the EHHO to optimize all of the BP parameters simultaneously, the weights and biases are sequentially encoded into a one-dimensional decision vector, as follows:

(5) $$\theta ={\left[vec{\left(W^{\left(1\right)}\right)}^{T},{\left(b^{\left(1\right)}\right)}^{T},vec{\left(W^{\left(2\right)}\right)}^{T},(b^{\left(2\right)})\right]}^{T}$$

For the adopted 2–6–1 network, the dimension of the optimization vector is as follows:

(6) D=6×2+6+6×1+1=25

Each decision variable is restricted to the interval [−3, 3]. Therefore, each individual q_i_∈R^D^ in the EHHO population represents one complete candidate set of BP weights and biases. In this notation, q_i_ is used exclusively for the optimization individual to avoid confusion with the physical variables used in Section 2.1 and Section 2.3.

Based on the conventional HHO algorithm [24], an elite-retention and mutation strategy inspired by the molting behavior of the Crayfish Algorithm [25] is introduced. At each iteration, the population is ranked according to the fitness value, and the top 10% of individuals are retained as the elite set. For a candidate individual, q_i_, the molting-inspired mutation is defined as follows:

(7) $$q_{i}^{mut}=q_{i}+\beta \cdot (ub-lb)\cdot (r-0.5)$$

where β=0.1 is the mutation coefficient, ub and lb denote the upper and lower search bounds, respectively, and r is a random vector whose elements are uniformly distributed in [0, 1].

To prevent the mutated solution from exceeding the prescribed search range, boundary handling is performed as follows:

(8) $$q_{i}^{mut}=\max[\min(q_{i}^{mut},ub),lb]$$

A greedy selection mechanism is subsequently employed as follows:

(9) $$q_{i}^{new}=\left\{\begin{array}{l} q_{i}^{mut},J(q_{i}^{mut})<J(q_{i})\\ q_{i},J(q_{i}^{mut})\geq J(q_{i}) \end{array}\right.$$

where the J function denotes the fitness measure, which is the root mean squared error (RMSE) of the BP neural network in this paper.

In addition, a vertical-crossover operation is introduced to alleviate the reduction in population diversity during the later optimization stage. Two candidate solutions are selected, and a new candidate solution is generated according to the following:

(10) $$q^{vc}=r\cdot q_{p}^{new}+(1-r)\cdot q_{q}^{new}$$

where $q_{p}^{new}$ and $q_{q}^{new}$ denote two selected candidate solutions. The crossover solution is retained only when its fitness is improved. The elite-retention, mutation, and vertical-crossover mechanisms are combined with the original HHO position-update strategy to maintain population diversity while preserving high-quality solutions.

The population size and maximum number of EHHO iterations are set to 50 and 200, respectively. After termination of the EHHO search, the best individual, $q_{best}$, is decoded into W^(1)^, b^(1)^, W^(2)^ and b^(2)^, which are used as the initial parameters of the BP network. The network is then fine-tuned using the Levenberg–Marquardt backpropagation algorithm, with a maximum of 1000 training epochs and a target error of 10^−8^. The resulting network is subsequently employed to estimate the nonlinear static calibration relationship between the blade-tip position and sensor voltage.

**Figure 1:**
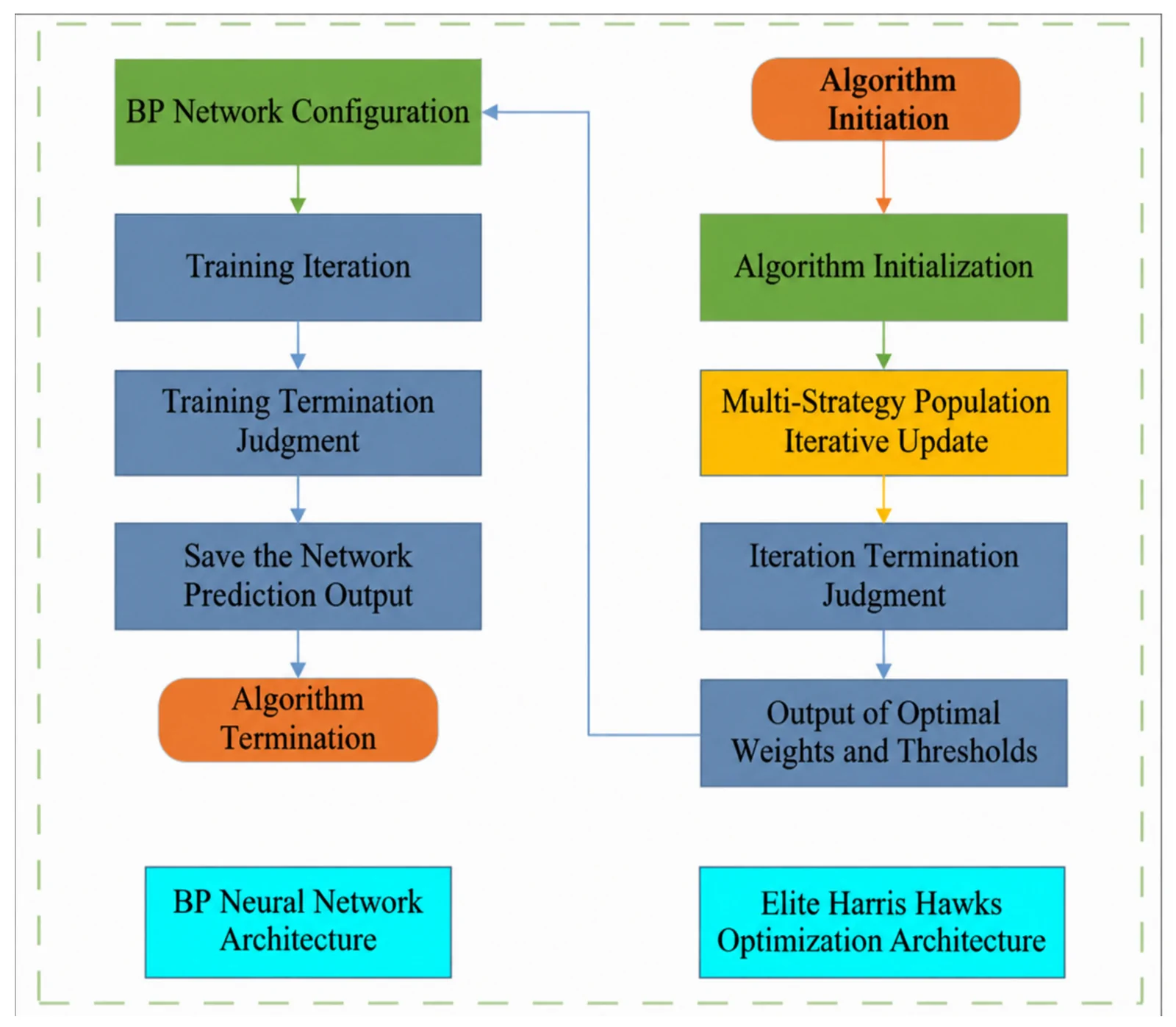
A framework for optimizing EHHO parameters in BP networks.

By means of the response estimation model based on the optimized BP neural network, the optimization process of this algorithm and the overall algorithm flow are illustrated in the following Figure 1. The nonlinear and asymmetric characteristics of the BTT voltage–position relationship make the optimization of the BP parameters critical for improving the approximation accuracy.

### 2.3. Method for Dynamic Sensor-Response Estimation Based on Waveform Mapping

Based on the static calibration relationship established in Section 2.1, the dynamic voltage response of the BTT sensor is estimated by combining the rigid-body blade-passing position with the circumferential blade-tip vibration displacement. The static calibration relationship obtained by the EHHO-BP model is expressed as Equation (2).

Since the static calibration is established in the angular domain, whereas the vibration information obtained from the BTT measurements is expressed as circumferential displacement, a coordinate transformation is introduced between these two representations. Let $R_{t}$ denote the rotational radius of the blade tip and s denote the circumferential blade-tip position. The relationship between s and α is as follows:

(11) $$s=R_{t}\alpha$$

where α is expressed in radians. Since the angular position in the static calibration experiment is expressed in arcminutes, the corresponding angular coordinate is calculated as follows:

(12) $$\alpha_{arc\min}=\frac{s}{R_{t}}\cdot \frac{180}{\pi }\cdot 60$$

Accordingly, the static voltage–angular-position relationship at the fixed radial clearance can be equivalently expressed in the circumferential displacement domain as follows:

(13) $$U=G_{c_{0}}(s)=F(c_{0},\alpha_{arc\min})$$

where $G_{c_{0}}(s)$ denotes the static voltage–circumferential-position mapping at the fixed blade-tip clearance, $c_{0}$.

Under constant-speed operation, the circumferential position of the blade tip in the absence of vibration is determined by the rigid-body rotation of the rotor. Taking the key-phase pulse as the reference for the blade-passing timing, the rigid-body angular position can be written as follows:

(14) $$\alpha_{r}(t)=\alpha_{0}+\Omega (t-t_{0})$$

where Ω is the rotational angular velocity, $t_{0}$ is the reference time determined from the key-phase signal, and $\alpha_{0}$ denotes the corresponding initial angular position. The rigid-body circumferential position is, therefore, as follows:

(15) $$s_{r}(t)=R_{t}\alpha_{r}(t)$$

Blade vibration introduces an additional circumferential displacement relative to the rigid-body blade-passing trajectory. For a general multi-harmonic vibration condition, the circumferential vibration displacement of the blade tip is expressed as follows:

(16) $$s_{v}(t)=\sum_{k=1}^{N_{h}}A_{k}\sin(2\pi f_{k}t+\varphi_{k})$$

where $N_{h}$ denotes the number of harmonic components, and $A_{k}$, $f_{k}$, and $\varphi_{k}$ represent the amplitude, vibration frequency, and initial phase of the k-th harmonic component, respectively. Here, $A_{k}$ and $s_{v}(t)$ are circumferential displacement quantities and are expressed in millimeters.

For BTT measurements, the blade vibration displacement is initially obtained as discrete circumferential displacement samples from the TOA information. Harmonic fitting is then used to reconstruct the continuous vibration displacement $s_{v}(t)$. Thus, the same displacement representation is used for both the subsequent simulations and the experimental BTT data processing.

The instantaneous circumferential position of the vibrating blade tip is obtained by superimposing the vibration displacement on the rigid-body position, as follows:

(17) $$s_{d}(t)=s_{r}(t)+s_{v}(t)$$

Substituting Equation (16) into Equation (17) gives the following:

(18) $$s_{d}(t)=s_{r}(t)+\sum_{k=1}^{N_{h}}A_{k}\sin(2\pi f_{k}t+\varphi_{k})$$

The corresponding instantaneous angular position is, therefore, as follows:

(19) $$\alpha_{d}(t)=\frac{s_{d}(t)}{R_{t}}=\alpha_{r}(t)+\frac{s_{v}(t)}{R_{t}}$$

Equation (19) explicitly establishes the conversion between the circumferential vibration displacement obtained from BTT and the angular-position variable used in the static calibration model.

**Figure 2:**
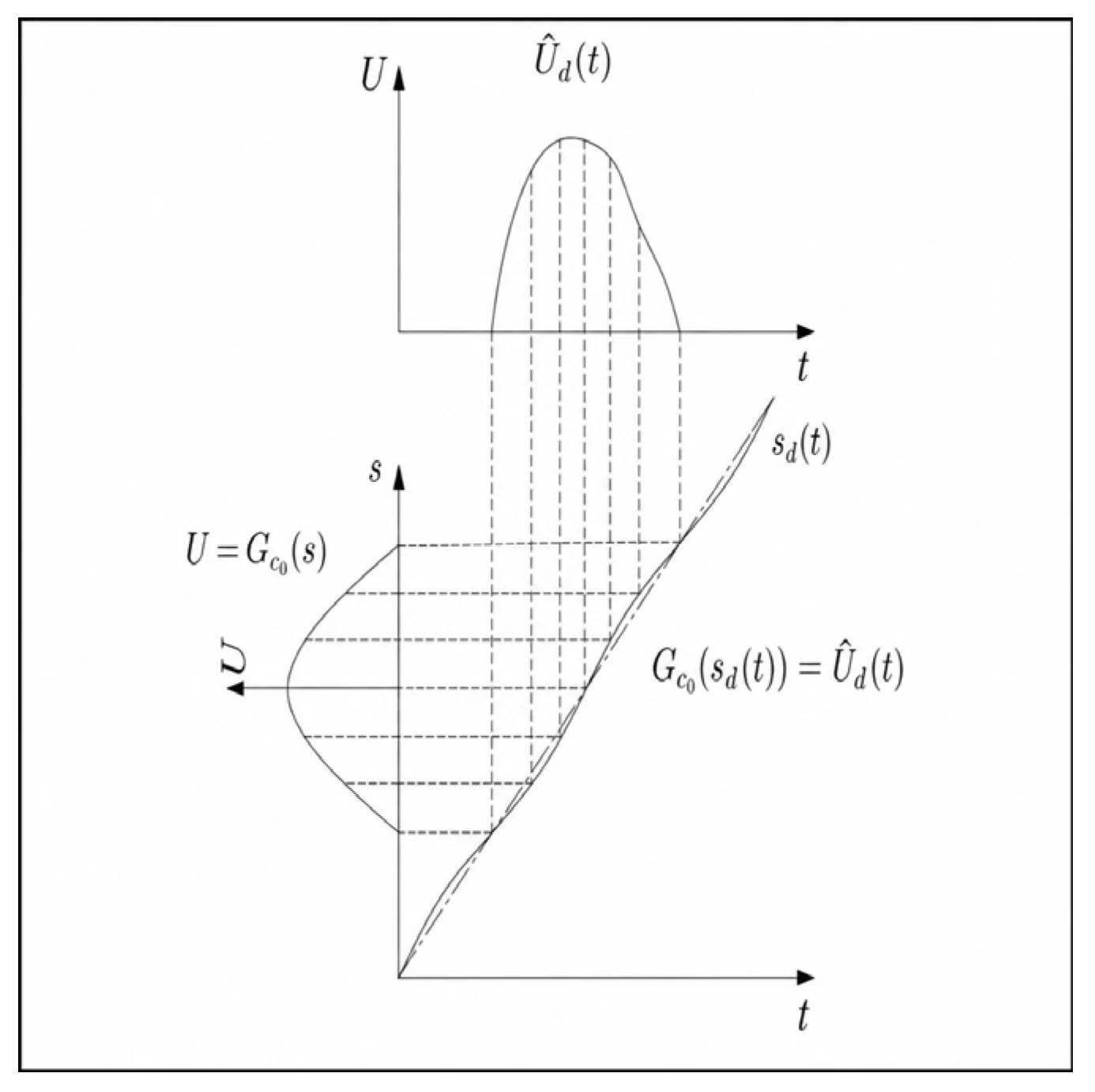
Schematic diagram of the waveform mapping-based dynamic response estimation method.

The waveform-mapping principle [20] is illustrated in Figure 2. Within the effective sensing region, it is assumed that voltage-equivalent points in the static calibration response and the dynamic sensor response correspond to the same instantaneous blade-tip position. Since the radial blade-tip clearance is fixed at $c_{0}$, the instantaneous dynamic voltage response is determined by the circumferential position, $s_{d}(t)$. Accordingly, the estimated dynamic voltage response is expressed as follows:

(20) $$U_{d}(t)=G_{c_{0}}[s_{d}(t)]=F\left[c_{0},\frac{s_{r}(t)+s_{v}(t)}{R_{t}}\cdot \frac{180}{\pi }\cdot 60\right]$$

When the instantaneous blade-tip position exceeds the effective calibration range of the sensor, the corresponding output is set to zero. Therefore, the complete waveform-mapping relationship can be expressed as follows:

(21) $$U_{d}(t)=\left\{\begin{array}{l} G_{c_{0}}[s_{d}(t)],s_{d}(t)\in S_{c_{0}}\\ 0,s_{d}(t)\notin S_{c_{0}} \end{array}\right.$$

where $S_{c_{0}}$ denotes the effective circumferential sensing interval corresponding to the fixed radial clearance, $c_{0}$.

Therefore, the proposed waveform-mapping procedure can be summarized as follows: the circumferential vibration displacement, $s_{v}(t)$, obtained either from the vibration model or reconstructed from the BTT measurements, is first superimposed on the rigid-body blade-passing position, $s_{r}(t)$, to obtain the instantaneous circumferential position, $s_{d}(t)$. This position is then converted into the corresponding angular coordinate and substituted into the static EHHO-BP calibration model at the fixed radial clearance, $c_{0}$, thereby obtaining the continuous dynamic voltage response, $U_{d}(t)$.

## 3. Simulation and Experimental Setup

### 3.1. Static Calibration Dataset and Model Evaluation Protocol

Static calibration data obtained under zero-speed conditions were used to construct and evaluate the calibration-response estimation models [22]. The radial blade-tip clearance ranged from 0.10 to 0.55 mm. To evaluate the generalization capability of the models at previously unseen clearances, the calibration data were divided according to complete clearance groups rather than randomly separating individual samples. The data corresponding to c = 0.15, 0.25, 0.35, and 0.45 mm were excluded from model training and used exclusively as independent test data, while the remaining clearance groups were used for model fitting and parameter selection.

The following five calibration-response estimation models were considered: conventional BP, HHO-BP, EHHO-BP, Gaussian RBF, and Kriging. All BP-based models adopted the network configuration, as described in Section 2.2. To evaluate the influence of stochastic initialization, BP, HHO-BP, and EHHO-BP were independently executed 30 times under the same dataset and parameter settings. RMSE, CVRMSE, MAE, R^2^, and training time were used as evaluation indices. The median values were used to characterize the representative model performance, whereas the mean and standard deviation over 30 independent runs were used to assess the overall prediction accuracy and run-to-run variability.

### 3.2. Dynamic Simulation Setup

Numerical simulations were conducted to evaluate the dynamic voltage-response estimation capability of the proposed waveform-mapping method under different blade vibration conditions. The radial blade-tip clearance was fixed at c_0_ = 0.1 mm, the sampling frequency was 5120 Hz, and the rotational angular velocity was 40π/3 rad/s. The continuous vibration displacement was generated according to the harmonic model described in Section 2.3 and subsequently superimposed on the rigid-body blade-passing position to obtain the instantaneous circumferential blade-tip position.

Three representative vibration conditions were considered. For the single-harmonic synchronous condition, the vibration amplitude was 1 mm, the excitation order was 6, and the initial phase was −π/10 rad. For the single-harmonic asynchronous condition, the same amplitude and initial phase were retained, while the excitation order was changed to 30.3. For the multi-harmonic condition, the excitation orders were [22, 35, 44.3], the amplitudes were [0.8, 0.5, 1] mm, and the initial phases were [−π/10, π/8, −π/6] rad.

For each vibration condition, the simulated circumferential vibration displacement was transformed into the corresponding dynamic sensor voltage using the static EHHO-BP calibration relationship and the waveform-mapping procedure described in Section 2.3.

### 3.3. Experimental Apparatus and Static Calibration Procedure

**Figure 3:**
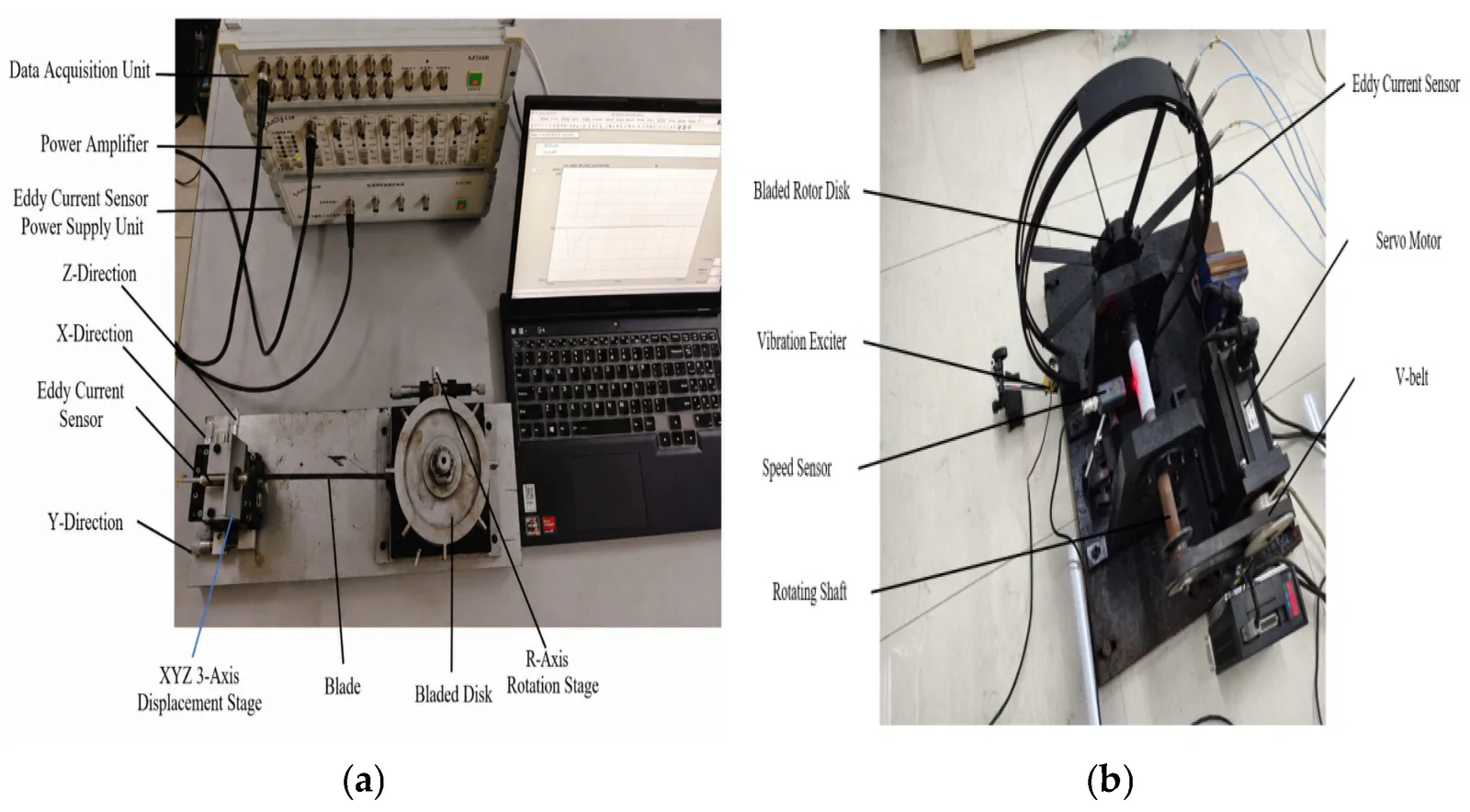
Experimental apparatus: (**a**) static calibration test device; (**b**) non-contact blade vibration measurement device.

The experimental platform consists of a static calibration system and a non-contact BTT vibration measurement system, as shown in Figure 3. As shown in Figure 3a, the static calibration system employs a TR81 eddy-current sensor and an RSP125-L R-axis calibration stage to obtain the voltage response at different radial blade-tip clearances and relative angular positions. The experimental setup (custom-built in our laboratory) was used for measurements. The main blade parameters are summarized in Table 1.

Static calibration measurements were conducted under zero-speed conditions. The radial blade-tip clearance was varied from 0.10 to 0.55 mm, and the sensor voltage was recorded at an angular-position interval of 2.3 arcmin. These measurements were used to establish the zero-speed voltage–position dataset required for EHHO-BP model’s training. Importantly, the calibration model established from this zero-speed dataset was subsequently retained unchanged during all rotating-condition experiments.

### 3.4. Dynamic BTT Measurement and Vibration Reconstruction Procedure

As shown in Figure 3b, dynamic blade vibration measurements were performed using the non-contact BTT system. Four BTT sensors were employed for blade-passing signal acquisition, and the key-phase signal was used to establish the rotational reference. The circumferential sensor positions were 0°, 16°, 29°, and 53°. DC-blocking processing was first applied to the measured voltage signals to remove the signal offset and facilitate the subsequent blade-arrival-time detection.

For each revolution, the blade TOA was extracted from the corresponding BTT voltage pulse, and the key-phase signal was used to determine the reference arrival time. The circumferential vibration displacement of the investigated blade was then calculated from the TOA deviation using the BTT processing procedure.

Since BTT provides discrete vibration-displacement samples at the sensor positions rather than a continuous waveform, harmonic fitting was employed to reconstruct the continuous blade vibration displacement, $s_{v}(t)$ [26]. The fitting procedure followed the harmonic vibration model described in Section 2.3. The reconstructed continuous displacement was subsequently used as the input to the waveform-mapping procedure for the dynamic voltage-response estimation.

### 3.5. Multi-Speed Experimental Protocol and Evaluation Metrics

To evaluate the applicability of the zero-speed static calibration relationship under rotating conditions, dynamic experiments were conducted at the following five constant rotational speeds: 400, 600, 800, 1200, and 1500 rpm. The original reference condition was 600 rpm, and four additional constant-speed conditions were introduced. The blade, sensor configuration, radial blade-tip clearance, excitation method, and data-acquisition system were kept unchanged across the five tests.

The EHHO-BP calibration model established exclusively from the zero-speed static calibration data was applied directly to all five rotating conditions without retraining or speed-dependent parameter correction. At each speed, multiple blade-passing voltage responses were aligned using the detected blade-arrival instants and averaged to obtain a representative measured voltage waveform.

At the same time, the TOA information obtained at each rotational speed was used to reconstruct the corresponding discrete blade-tip vibration displacement. Harmonic fitting was applied independently at each speed to obtain a continuous circumferential vibration-displacement waveform. The fitted displacement was combined with the rigid-body blade-passing position and substituted into the zero-speed EHHO-BP calibration relationship according to the waveform-mapping procedure in Section 2.3, yielding the reconstructed dynamic voltage response.

Before the quantitative waveform comparison, temporal normalization was applied to place the measured and reconstructed responses on a common time coordinate. The detected blade-arrival instant was taken as the temporal reference, and the two effective observation intervals were linearly mapped to a common normalized interval; the reconstructed voltage was then interpolated onto the measured sampling grid. No voltage-amplitude normalization was applied. Correlation, R^2^, and NRMSE were calculated on this common time grid, while the peak-voltage error is unaffected by the temporal mapping. The FWHM error was calculated after normalization and, therefore, represents the residual relative difference in waveform width rather than a difference caused only by unequal observation-window lengths.

For each rotational-speed condition, the reconstructed waveform was compared with the measured averaged waveform. The correlation coefficient, R^2^, NRMSE, peak-voltage error, and full width at half maximum (FWHM) error were used to evaluate the waveform morphology, overall reconstruction accuracy, amplitude consistency, and temporal-width consistency. The variation of these indices with rotational speed was used to assess the quasi-static transfer assumption and the experimentally supported operating range of the proposed method.

## 4. Results and Discussion

### 4.1. Static Calibration and Model Comparison

**Figure 4:**
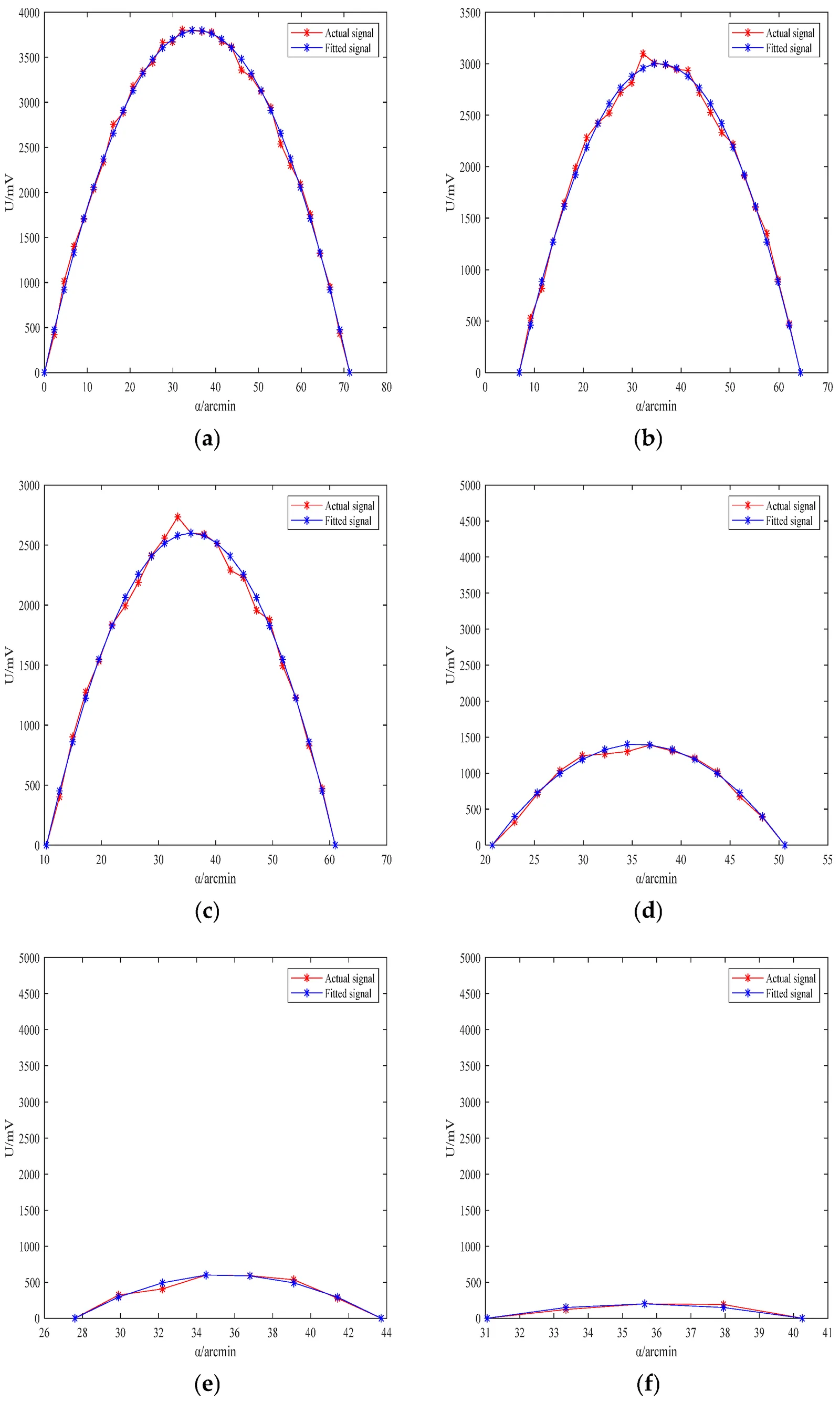
Static calibration data at blade-tip clearances of 0.1–0.55 mm: (**a**) fitted experimental data at 0.1 mm; (**b**) fitted experimental data at 0.2 mm; (**c**) fitted experimental data at 0.3 mm; (**d**) fitted experimental data at 0.4 mm; (**e**) fitted experimental data at 0.5 mm; (**f**) fitted experimental data at 0.55 mm.

The measured static calibration responses at different radial blade-tip clearances are presented in Figure 4. The sensor voltage exhibits a pronounced nonlinear dependence on both the radial clearance and relative blade-tip position, providing the experimental basis for constructing the calibration-response estimation models.

To evaluate the estimation accuracy and generalization capability of different calibration-response models, five methods—standard BP, HHO-BP, EHHO-BP, Gaussian radial basis function network (Gaussian RBF), and Kriging—were compared using the same static calibration dataset and identical training and test-clearance groups. Before model training, the original sensor voltage, *U_r_*, was transformed as follows:

(22) $$U^{*}=\left|U_{r}+5000\right|$$

where U* denotes the processed calibration-response voltage. This transformation converts the original negative voltage response into a positive response while preserving the relative waveform variation.

To avoid overly optimistic results caused by randomly separating individual data points, the calibration data were divided according to complete blade-tip-clearance groups. The data corresponding to c = 0.15, 0.25, 0.35, 0.45 mm were excluded from model training and used exclusively as independent test data. The remaining clearance groups were used for parameter selection and model fitting. Therefore, the comparison primarily evaluates the ability of each model to estimate the complete sensor-response waveform at previously unseen blade-tip clearances.

**Figure 5:**
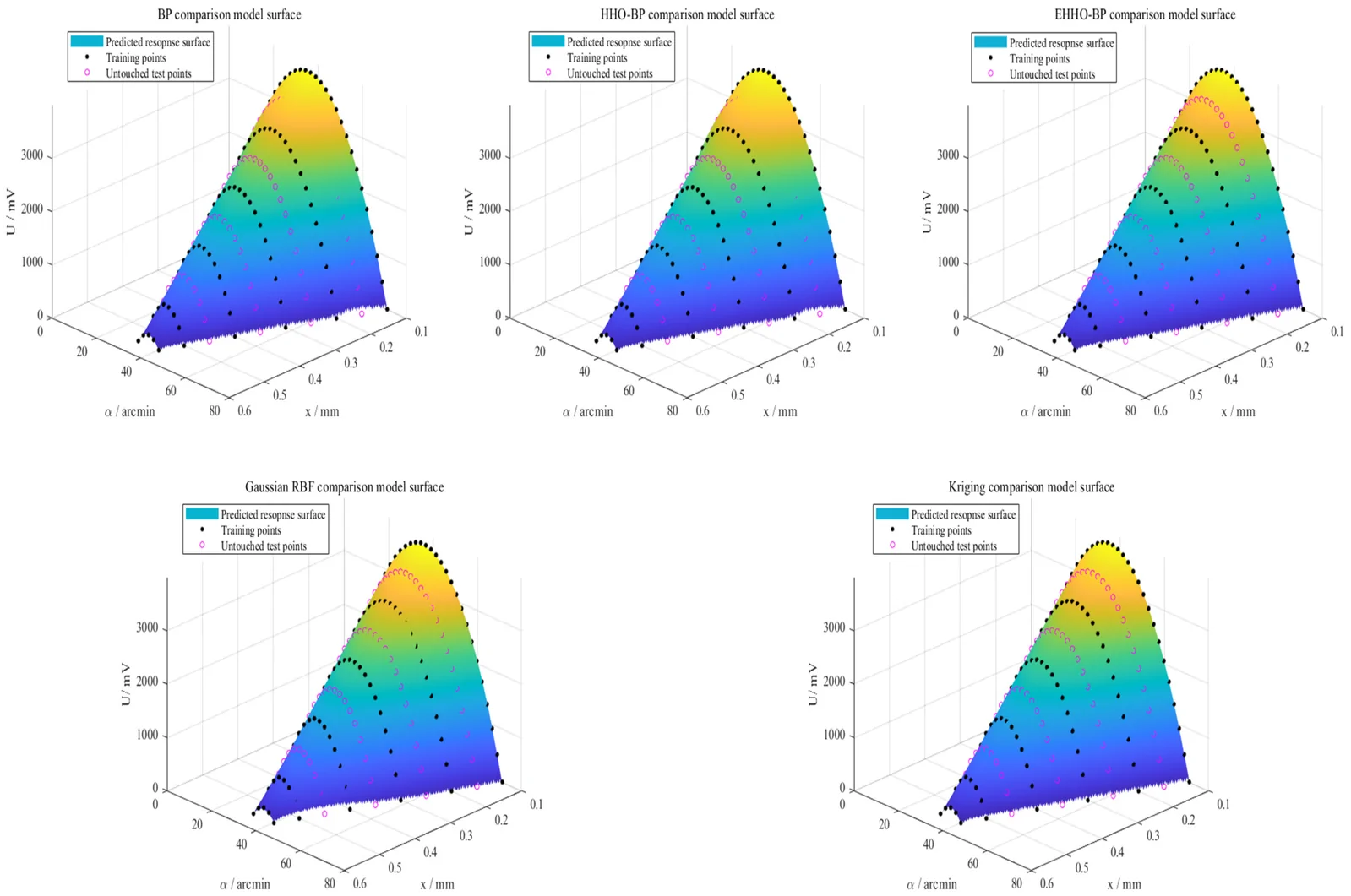
Estimated static calibration response surfaces obtained using different models.

Figure 5 compares the static calibration response surfaces estimated by the standard BP, HHO-BP, EHHO-BP, Gaussian RBF, and Kriging models. The black points denote the calibration samples used for model training, whereas the magenta hollow points correspond to the four blade-tip-clearance groups excluded from the training and retained exclusively for independent testing. All five models capture the principal variation characteristics of the static calibration response. As the blade-tip clearance increases, the maximum response voltage gradually decreases, and the effective angular range over which the blade interacts with the sensor becomes narrower. In addition, the estimated response waveforms exhibit an approximately symmetric and unimodal shape with respect to the central angular position.

Compared with the standard BP model, HHO-BP provides improved agreement with the calibration samples, indicating that HHO-based parameter optimization enhances the approximation capability of the BP network. The EHHO-BP model further improves the surface fitting, particularly near the response peak and in the large-clearance region, and exhibits smoother and more consistent variation across the calibration domain. This improvement demonstrates the effectiveness of the proposed elite-retention and vertical-crossover strategies in enhancing the optimization and generalization performance of HHO-BP. In comparison with the other surrogate models, Gaussian RBF captures the overall response trend but shows relatively larger deviations in the sparsely sampled large-clearance region, whereas Kriging provides a smooth and accurate response surface. Overall, EHHO-BP shows improved fitting consistency over BP and HHO-BP and provides competitive response-surface estimation compared with Gaussian RBF and Kriging.

**Figure 6:**
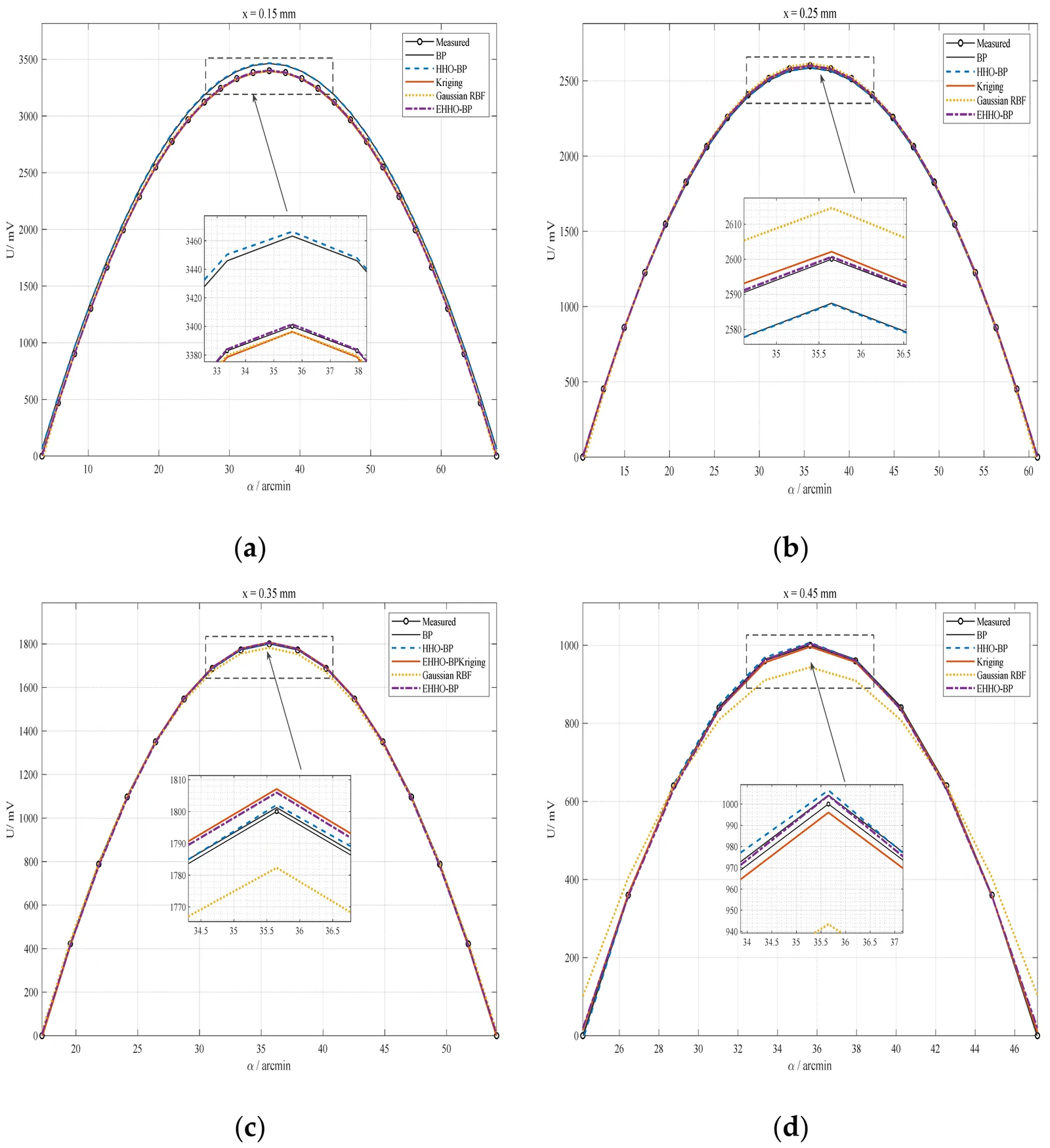
Comparison between the measured and estimated calibration-response waveforms at previously unseen blade-tip clearances: (**a**) c = 0.15 mm; (**b**) c = 0.25 mm; (**c**) c = 0.35 mm; (**d**) c = 0.45 mm.

To further examine the differences among the models, Figure 6 compares the estimated waveforms at the four untouched blade-tip clearances. The enlarged peak regions reveal clearer differences that are difficult to distinguish from the complete waveforms. The standard BP model shows relatively evident deviations from the measured peak responses. Although HHO-based optimization improves the BP prediction, residual peak errors remain at several test clearances. By comparison, EHHO-BP consistently produces predictions closer to the measured data, demonstrating that the introduced elite-retention and vertical-crossover strategies further improve the optimization performance of the original HHO-BP model. Kriging also provides close agreement with the measured waveforms, whereas Gaussian RBF exhibits larger peak deviations as the clearance increases, especially at c = 0.35 and 0.45 mm. These results indicate that the advantage of EHHO-BP is mainly reflected in the accurate reconstruction of the peak-response region and its stable generalization across unseen clearance conditions.

**Figure 7:**
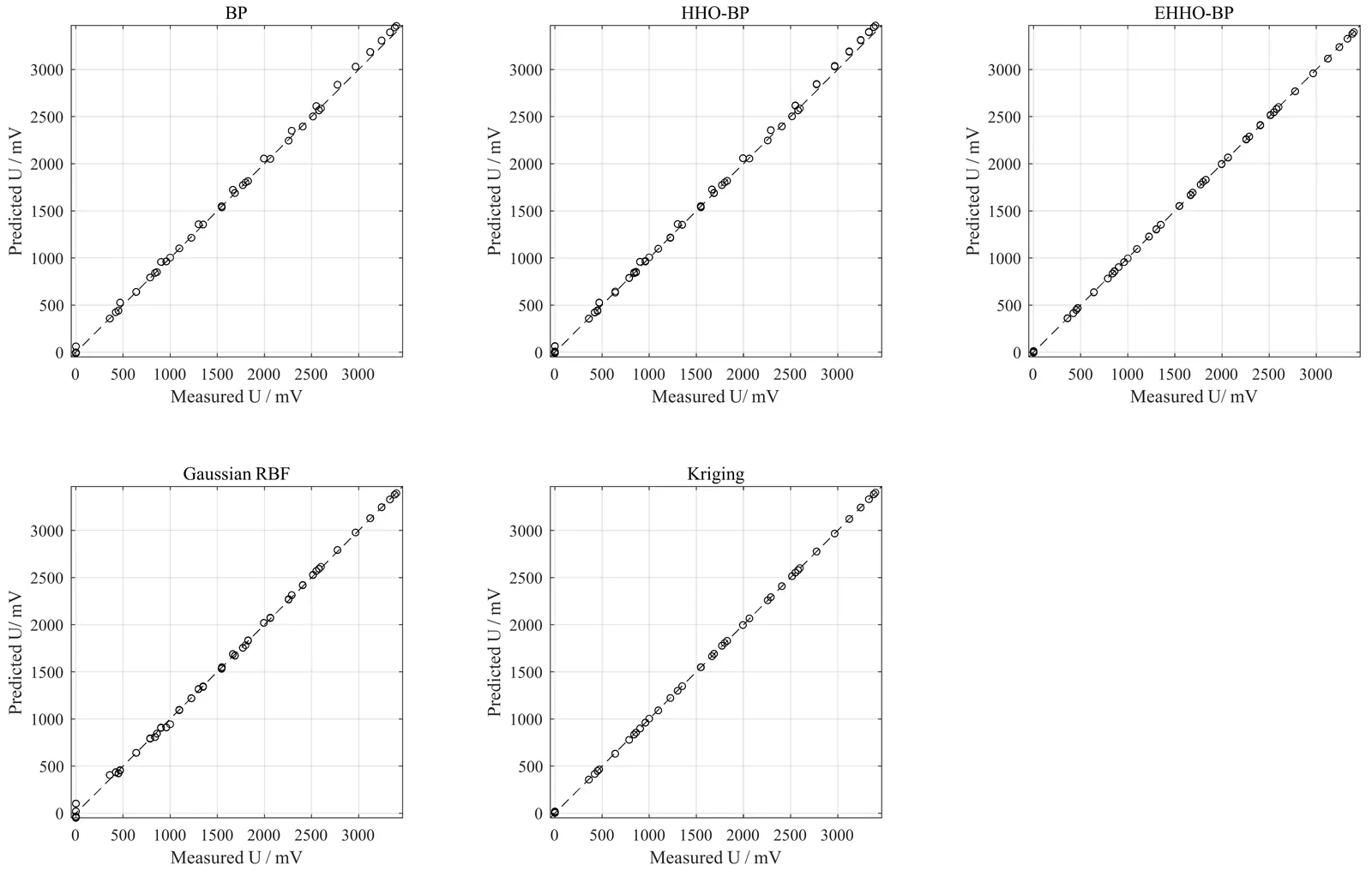
Predicted versus measured calibration responses for the untouched test samples.

Figure 7 compares the predicted and measured responses of the five models. All predictions are generally distributed along the ideal line, indicating good overall agreement with the measured data. Compared with the standard BP model, HHO-BP reduces the prediction dispersion, while EHHO-BP further concentrates the predicted points around the ideal line, demonstrating the effectiveness of the proposed EHHO strategy in improving the prediction and generalization capability of BP. Kriging also shows close agreement with the measured responses, whereas Gaussian RBF exhibits relatively larger deviations for some low-response samples. These results further confirm the improved prediction consistency of EHHO-BP over BP and HHO-BP.

The quantitative comparison is presented in Table 2 and Table 3. Table 2 reports the median values of the evaluation metrics for each calibration-response estimation model, representing their typical prediction performance. Table 3 presents the mean ± standard deviation obtained from 30 independent runs of BP, HHO-BP, and EHHO-BP, where the mean characterizes the overall prediction accuracy and the standard deviation reflects the variability caused by stochastic initialization.

As shown in Table 2, EHHO-BP achieves the best median prediction performance, with an RMSE of 8.92 mV, a CVRMSE of 0.56%, an MAE of 6.34 mV, and an R^2^ of 0.99982. These errors are lower than those of conventional BP, HHO-BP, Gaussian RBF, and Kriging, indicating the improved approximation capability of the proposed method. The results in Table 3 further confirm this improvement over repeated independent runs. As shown in Table 3, the RMSE decreased from 31.82 ± 18.46 mV for BP to 18.47 ± 7.62 mV for HHO-BP and further to 11.84 ± 5.27 mV for EHHO-BP. A similar trend was observed for the MAE, which decreased from 22.71 ± 13.25 mV to 13.38 ± 5.54 mV and 8.57 ± 3.86 mV, respectively. In addition, the lower standard deviations obtained by EHHO-BP indicate reduced sensitivity to random initialization and improved prediction stability. Although EHHO-BP requires a longer training time because of the additional optimization operations, its computational cost remains acceptable for offline static calibration. Overall, the comparisons with BP and HHO-BP demonstrate that the proposed EHHO strategy improves both the prediction accuracy and robustness of the BP-based calibration-response model. More importantly, the direct comparison among BP, HHO-BP, and EHHO-BP confirms that the performance improvement originates from the proposed EHHO optimization strategy rather than from the BP network architecture alone.

### 4.2. Simulation Results of Dynamic Voltage-Response Estimation

**Figure 8:**
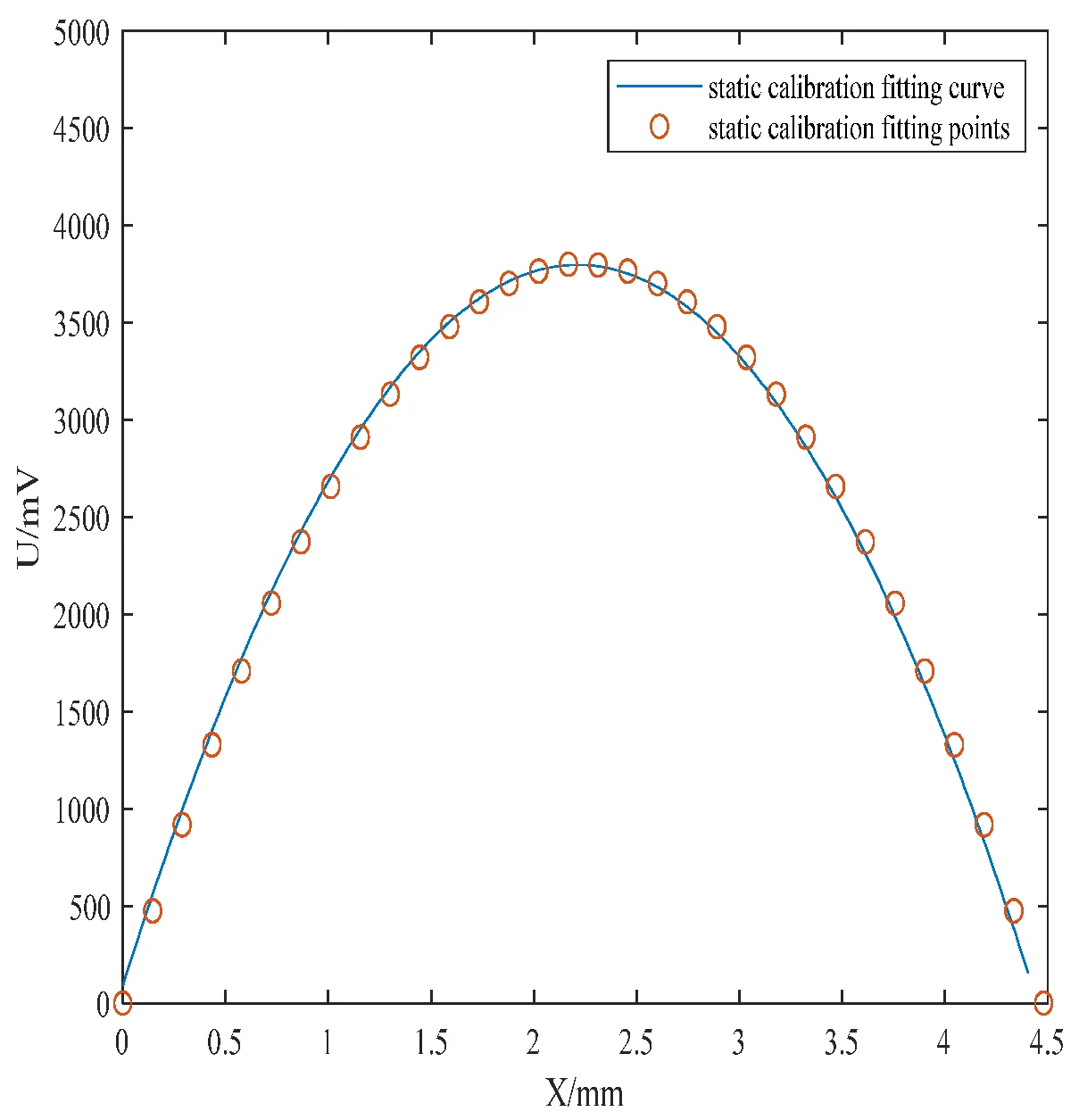
Fitting plot of the relationship between rotational rigid displacement and voltage under static calibration.

**Figure 9:**
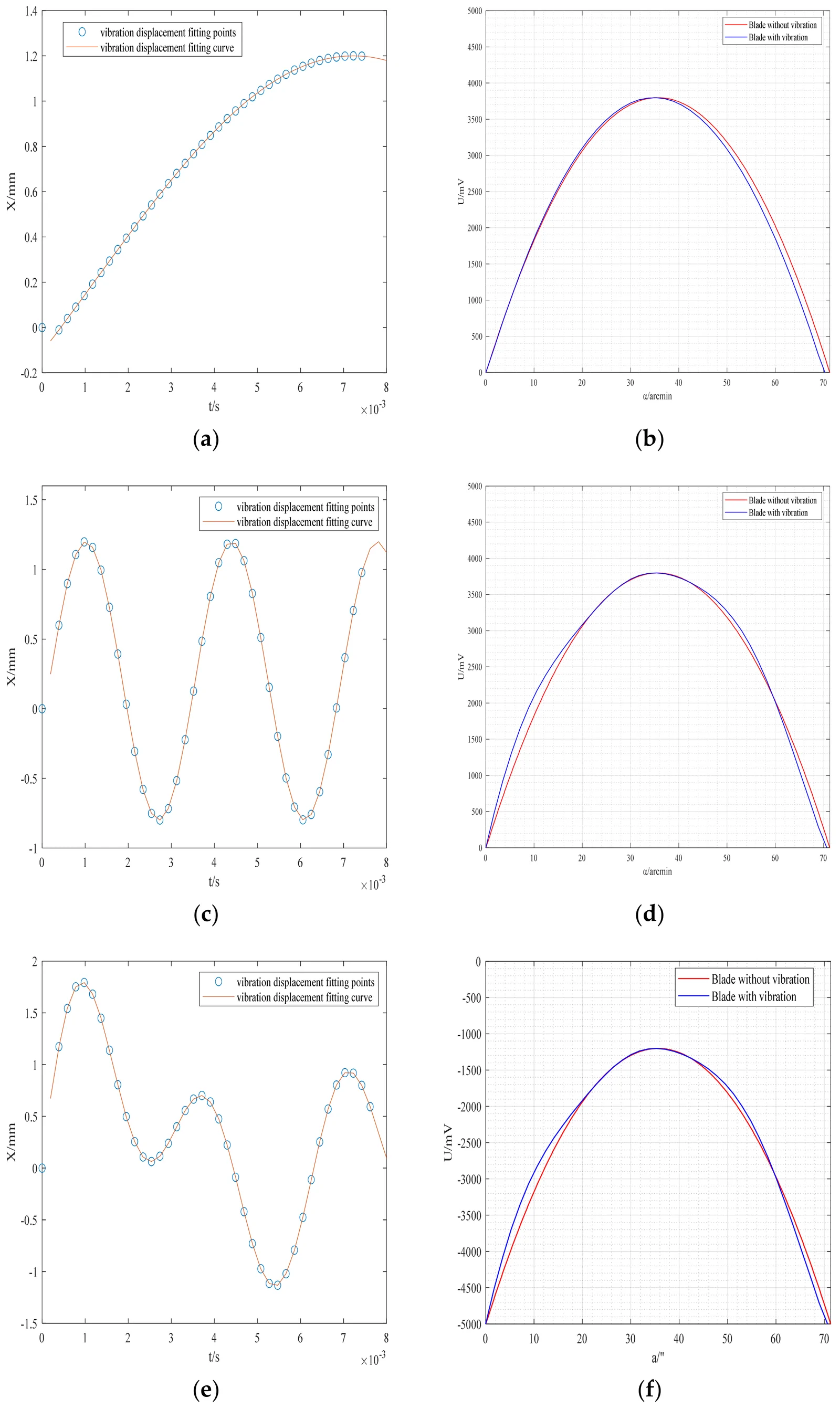
Estimated sensor-voltage response from simulated blade vibration displacement: (**a**) single-harmonic synchronous vibration displacement; (**b**) estimated dynamic voltage response; (**c**) single-harmonic asynchronous vibration displacement; (**d**) estimated dynamic voltage response; (**e**) multi-harmonic vibration displacement; (**f**) estimated dynamic voltage response.

Figure 8 presents the static voltage–position mapping used for the subsequent dynamic-response simulations. Figure 9 summarizes the simulated circumferential blade-tip vibration displacement and the corresponding estimated dynamic voltage responses under synchronous single-harmonic, asynchronous single-harmonic, and multi-harmonic vibration conditions.

#### 4.2.1. Single-Harmonic Blade Vibration Model

Figure 9 presents the simulated blade-tip vibration displacement and the corresponding dynamic sensor-voltage responses estimated using the waveform-mapping method under different vibration conditions. For a single-harmonic vibration condition, the blade-tip displacement is modeled as a harmonic response with the corresponding excitation order, with the vibration equation given in (16), where the amplitude A_0_ = 1 mm, excitation order Ne = 6, and initial phase is −π/10 rad. Figure 9a presents part of the blade-vibration displacement data. Figure 9b shows the estimated dynamic voltage response obtained from the waveform mapping relationship using the simulated vibration displacement, the comparison illustrates the influence of blade vibration displacement on the estimated sensor-voltage response. Meanwhile, it is evident that phase variation characteristics in the voltage response caused by blade vibration displacement can be observed. For single-harmonic asynchronous vibration of the blade, the same parameters as above are retained, while the excitation order is adjusted to 30.3. The corresponding data comparisons are presented in Figure 9c,d.

#### 4.2.2. Multi-Harmonic Blade Vibration Model

The prerequisites remain the same, the dynamic sensor-response estimation under a three-harmonic vibration condition is investigated in Figure 9e,f, where the excitation orders are [22, 35, 44.3], the amplitudes are [0.8, 0.5, 1] mm, and the initial phases are [−π/10, π/8, −π/6] rad.

Compared with the single-harmonic cases, the multi-harmonic displacement produces a more irregular voltage-response waveform, reflecting the combined influence of multiple vibration components on the instantaneous blade-tip position.

### 4.3. Experimental Vibration-Displacement Reconstruction at the Reference Speed

**Figure 10:**
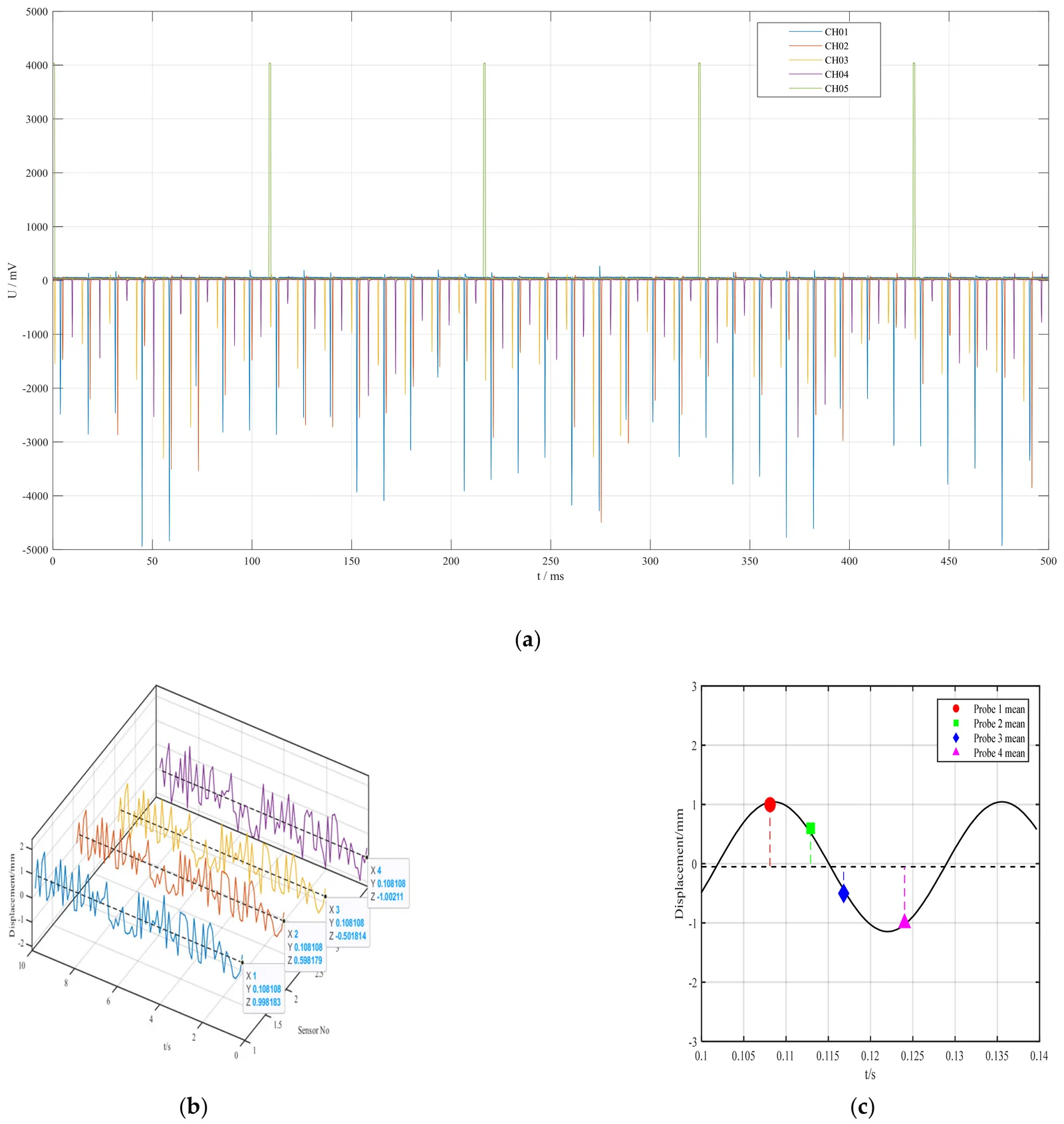
Experimental measurement and blade vibration displacement reconstruction using the BTT system: (**a**) raw BTT voltage signals and key-phase reference signal; (**b**) reconstructed blade vibration displacement based on TOA measurements; (**c**) harmonic fitting of blade vibration displacement using Liu’s method.

Figure 10 presents a representative BTT measurement and the corresponding blade-vibration displacement reconstruction at the reference rotational speed of 600 rpm. Figure 10a shows the simultaneously acquired BTT voltage signals and key-phase reference signal. Based on the detected TOA information, the circumferential vibration displacement of the investigated blade was reconstructed at the four sensor positions, as shown in Figure 10b. The discrete BTT displacement samples were subsequently fitted using the harmonic model [26], yielding the continuous vibration-displacement waveform shown in Figure 10c. The fitted displacement preserves the dominant periodic variation observed in the BTT measurements and provides the continuous motion input required by the waveform-mapping procedure. The black solid line represents the fitting curve, and the black dotted line denotes the baseline.

### 4.4. Dynamic Voltage-Response Reconstruction at the Reference Speed

Using the continuous blade vibration displacement reconstructed from the 600 rpm BTT measurements, the dynamic sensor-voltage response was estimated with the zero-speed EHHO-BP calibration model and the waveform-mapping procedure. The reconstructed response was then compared with the experimentally measured BTT voltage waveform to assess the consistency of the proposed mapping under the reference rotating condition.

**Figure 11:**
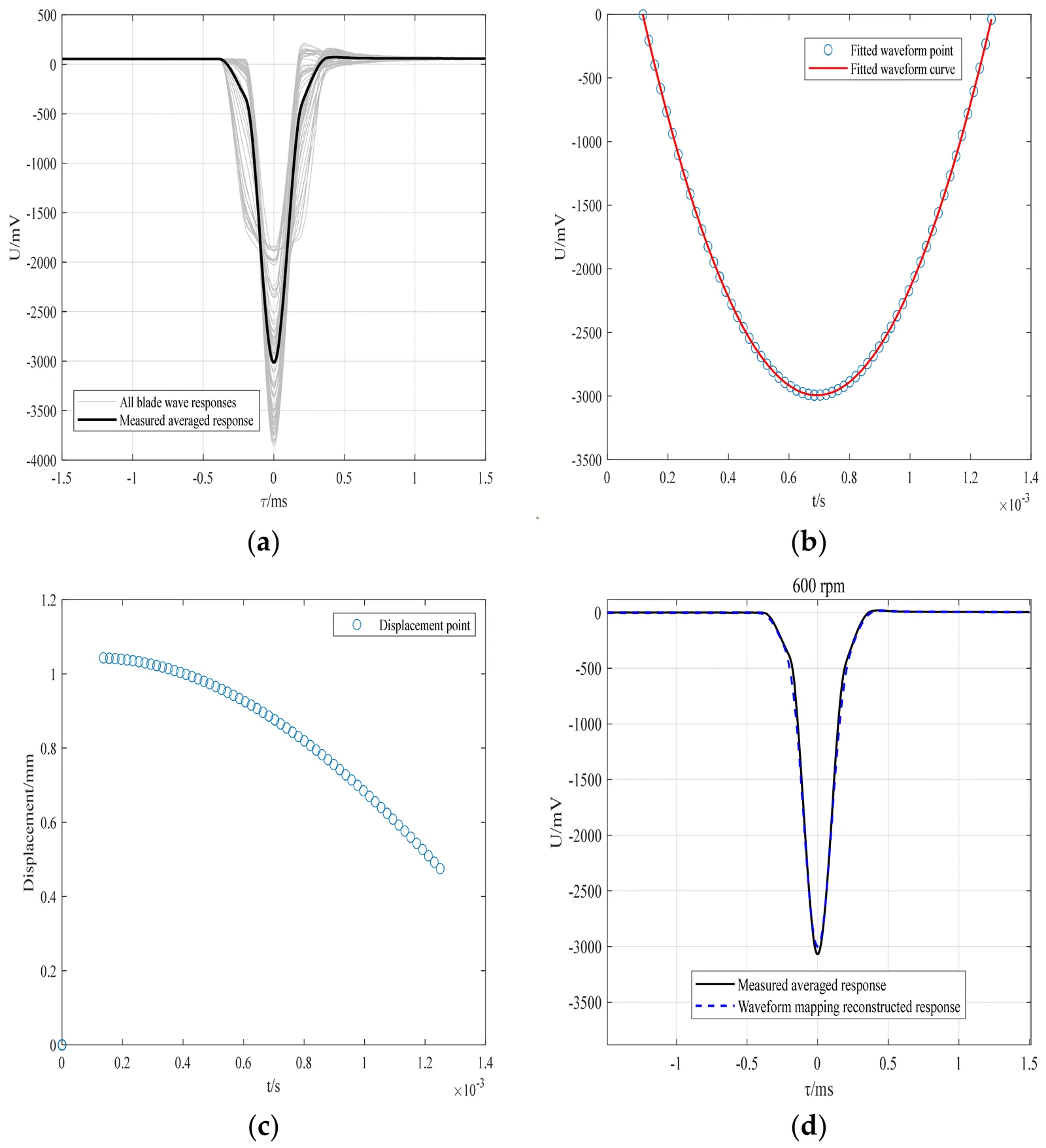
Experimental validation of dynamic voltage estimation based on waveform mapping: (**a**) multi-revolution averaged BTT voltage response; (**b**) waveform-mapped dynamic voltage response; (**c**) correspondence between reconstructed voltage and blade vibration displacement; (**d**) comparison between the estimated dynamic voltage response and the experimentally averaged BTT response.

As shown in Figure 11a, blade-passing voltage signals from multiple revolutions were aligned according to the detected blade-arrival instants and averaged to obtain a representative measured response. Averaging reduces random measurement fluctuations while preserving the repeatable waveform characteristics associated with the blade passage.

The continuous blade vibration displacement obtained by harmonic fitting was combined with the static voltage–position-calibration relationship to reconstruct the dynamic voltage waveform, as shown in Figure 11b. Figure 11c illustrates the pointwise correspondence between the reconstructed voltage and the blade vibration displacement. The procedure, therefore, converts the BTT-derived motion trajectory into a continuous sensor-domain waveform over the blade-passage interval.

The reconstructed voltage–displacement correspondence in Figure 11c should be interpreted as a continuous sensor-domain representation generated from BTT-derived displacement rather than as an independent source of vibration information. Each reconstructed voltage sample is determined by the instantaneous blade-tip position and the static calibration mapping.

This representation extends the conventional TOA-based description from discrete arrival-time deviations to a continuous voltage-domain waveform that can be compared directly with the measured probe response.

Figure 11d compares the reconstructed waveform with the measured averaged response at 600 rpm. The two responses show close agreement in peak amplitude and overall waveform morphology, supporting the use of the zero-speed calibration relationship at the reference rotating condition. Before comparison, the temporal-normalization procedure defined in Section 3.5 was applied to align the two responses on a common time coordinate without modifying the voltage amplitude; the same procedure was used for the subsequent multi-speed evaluation.

### 4.5. Multi-Speed Experimental Validation of the Quasi-Static Assumption

To evaluate whether the zero-speed static calibration relationship remains transferable over a broader range of rotational speeds, the proposed waveform-mapping method was tested at 400, 600, 800, 1200, and 1500 rpm. The same EHHO-BP calibration model obtained from zero-speed data was used for all conditions without retraining or speed-dependent parameter correction.

**Figure 12:**
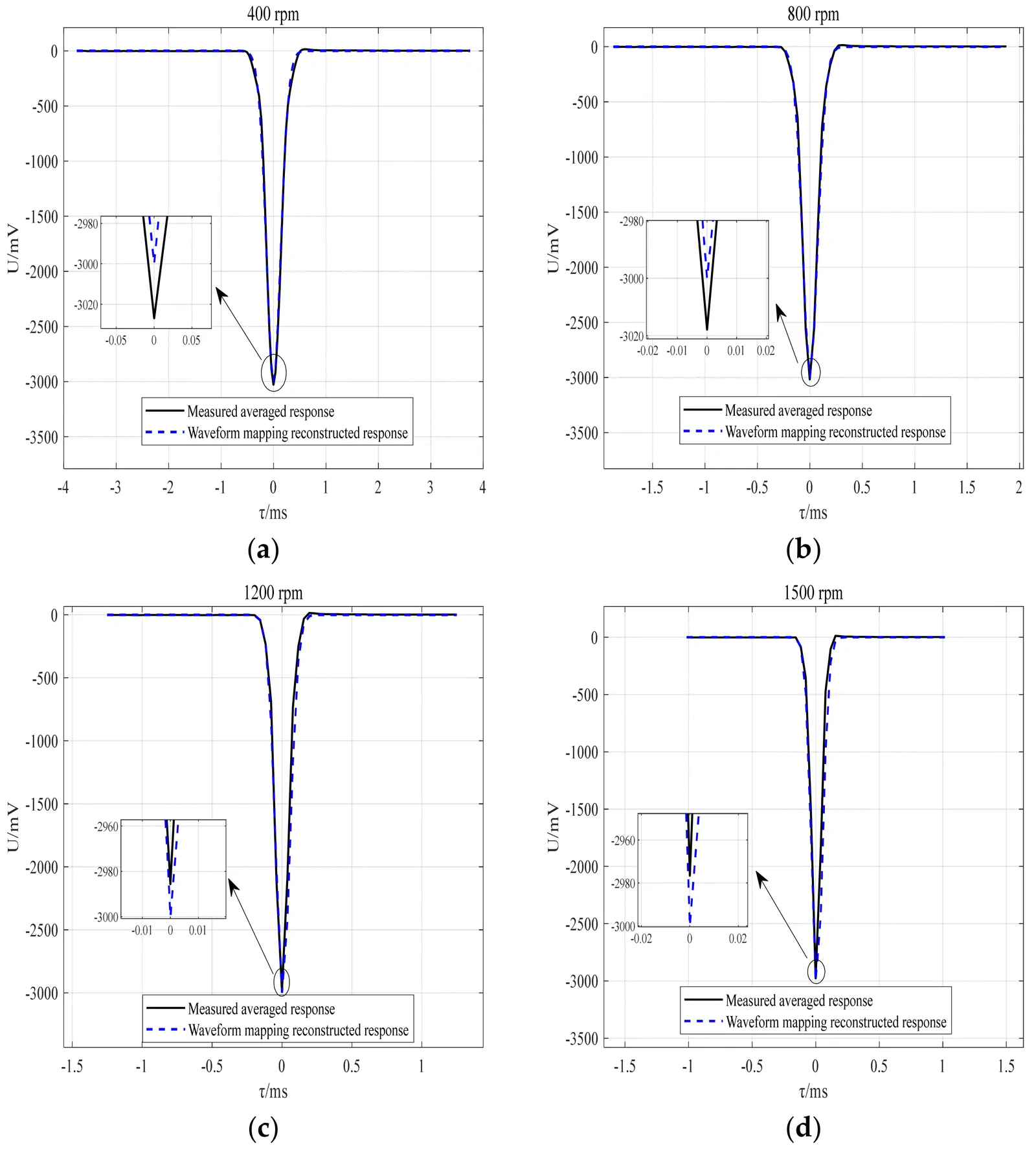
Comparison between the measured averaged and waveform-mapping-reconstructed voltage responses at different rotational speeds: (**a**) 400 rpm; (**b**) 800 rpm; (**c**) 1200 rpm; (**d**) 1500 rpm.

The 600 rpm comparison is presented in Figure 11d, while Figure 12 shows the corresponding comparisons at the four additional speeds of 400, 800, 1200, and 1500 rpm. For each operating condition, the reconstructed waveform was generated from the vibration displacement independently reconstructed from the TOA measurements at that speed. Table 4 summarizes the quantitative comparison.

The quantitative results show strong agreement at low-to-moderate rotational speeds. At 400, 600, and 800 rpm, the correlation coefficients are 0.9989, 0.9982, and 0.9945, respectively, while R^2^ remains between 0.996 and 0.997. Over the same range, NRMSE does not exceed 1.203, the peak-voltage error remains within 20–21 mV in magnitude, and the FWHM error ranges from 1.469% to 6.827%. These results indicate that the zero-speed calibration relationship retains good waveform-transfer capability in this speed range.

At 1200 rpm, the correlation coefficient remains high at 0.9926, but R^2^ decreases to 0.980 and NRMSE increases to 2.443. The FWHM error also increases to 10.799%, whereas the peak-voltage error is −18 mV. At 1500 rpm, the correlation coefficient and R^2^ decrease further to 0.9868 and 0.952, respectively, and NRMSE increases to 3.750. The peak-voltage error is −25 mV, while the FWHM error reaches 22.662%.

The speed-dependent trend is, therefore, more pronounced in the temporal width of the response than in its peak amplitude. The increasing FWHM error indicates that the quasi-static mapping becomes less accurate in reproducing the detailed temporal waveform as the rotational speed increases, even though the principal waveform shape and peak level remain reasonably consistent over the tested range. Accordingly, the present experiments support the quasi-static assumption most strongly at low-to-moderate speeds and reveal a progressive limitation at higher speeds.

The progressive deterioration in temporal consistency at higher speeds may be related to dynamic effects that are not represented in the zero-speed calibration relationship. As the rotational speed increases, the blade-passing duration becomes shorter and the probe response contains higher temporal-frequency components. The finite bandwidth and phase response of the eddy-current sensor, signal-conditioning circuit, and acquisition chain may, therefore, introduce increasing temporal lag or pulse-shape distortion. In addition, the electromagnetic response of an eddy-current probe is frequency-dependent, so rapid blade passage may cause departures from the quasi-static response obtained at zero speed. These effects provide a plausible explanation for the stronger deterioration in FWHM than in peak amplitude. The present experiments do not isolate these individual mechanisms, and a dedicated frequency-response characterization will be investigated in future work.

The present validation uses vibration displacement reconstructed from the same BTT measurement system that provides the voltage waveform used for comparison. Therefore, the experiment is primarily an assessment of transferability and waveform consistency rather than a fully independent validation of blade displacement. Future work should introduce an independent displacement reference and investigate speed-dependent probe dynamics to further separate sensor-dynamic effects from BTT reconstruction uncertainty.

## 5. Conclusions

This study proposes a dynamic voltage-response estimation framework that combines EHHO-BP static calibration with waveform mapping for BTT sensors. The main conclusions are as follows:

The EHHO-BP model improves the estimation of the nonlinear static calibration relationship. On the independent clearance test groups, the proposed model achieves a median RMSE of 8.92 mV, CVRMSE of 0.56%, MAE of 6.34 mV, and R^2^ of 0.99982, while repeated-run results also show lower prediction error and variability than conventional BP and HHO-BP.

By superimposing BTT-derived circumferential vibration displacement on the rigid-body blade-passing trajectory and mapping the instantaneous position through the static EHHO-BP calibration relationship, the method produces a continuous sensor-domain voltage representation. Single-harmonic, asynchronous, and multi-harmonic simulations demonstrate the mapping behavior, and the 600 rpm experiment shows close agreement between the reconstructed and measured averaged voltage responses.

Multi-speed experiments at 400, 600, 800, 1200, and 1500 rpm further assess the quasi-static transfer assumption using the same zero-speed calibration model without retraining. The correlation coefficient remains between 0.9868 and 0.9989 and R^2^ between 0.952 and 0.997 across the tested range. However, the FWHM error increases from 1.469% at 400 rpm to 22.662% at 1500 rpm, showing that temporal waveform consistency deteriorates progressively as rotational speed increases. The current results, therefore, support the proposed mapping most strongly in the low-to-moderate speed range and identify high-speed temporal distortion as an important limitation for further study.

The reconstructed voltage waveform should be regarded as a sensor-domain representation derived from BTT displacement information rather than an independent additional measurement. Future work will focus on independent displacement-reference validation, explicit characterization of speed-dependent sensor dynamics, and extension of the calibration model to a wider range of operating conditions.

## Figures and Tables

**Table 1:** Blade properties.

Blade Configuration	Value
Blade count	8
Blade radial length	161 mm
Blade width	20 mm
Blade thickness	2 mm
Blade material	Steel 45
Rotor radius	56 mm

**Table 2:** Median of the prediction performance for each calibration-response estimation model.

Model	RMSE	CVRMSE	MAE	R^2^	Training Time
BP	24.86	1.56	17.92	0.9987	3.42
HHO-BP	16.74	1.05	12.08	0.99935	4.21
EHHO-BP	8.92	0.56	6.34	0.99982	7.01
RBF	25.91	1.63	18.47	0.99931	1.24
Kriging	10.76	0.68	7.83	0.99973	2.56

**Table 3:** Mean ± standard deviation of the prediction performance over 30 independent runs.

Model	RMSE	CVRMSE	MAE	R^2^	Training Time
BP	31.82 ± 18.46	2 ± 1.16	22.71 ± 13.25	0.9974 ± 0.0031	3.49 ± 0.11
HHO-BP	18.47 ± 7.62	1.16 ± 0.48	13.38 ± 5.54	0.9991 ± 0.00072	4.25 ± 0.15
EHHO-BP	11.84 ± 5.27	0.74 ± 0.33	8.57 ± 3.86	0.9996 ± 0.00038	7.31 ± 0.18

**Table 4:** Quantitative error metrics for multi-speed dynamic voltage–response reconstruction.

Speed (rpm)	Correlation	R^2^	Peak Error (mV)	NRMSE	FWHM Error (%)
400	0.9989	0.997	20	0.799	1.469
600	0.9982	0.997	21	1.203	6.370
800	0.9945	0.996	20	1.079	6.827
1200	0.9926	0.980	−18	2.443	10.799
1500	0.9868	0.952	−25	3.750	22.662

## Data Availability

The data used to support the findings of this paper are available from the corresponding author upon request. The data are not publicly available due to privacy.
